# Brain-Derived Neurotrophic Factor Attenuates Septic Myocardial Dysfunction via eNOS/NO Pathway in Rats

**DOI:** 10.1155/2017/1721434

**Published:** 2017-07-09

**Authors:** Ni Zeng, Junmei Xu, Weifeng Yao, Suobei Li, Wei Ruan, Feng Xiao

**Affiliations:** ^1^Department of Anesthesiology, The Second Xiangya Hospital, Central South University, Changsha, Hunan 410011, China; ^2^Department of Anesthesiology, Third Affiliated Hospital, Sun Yat-sen University, Guangzhou, Guangdong 510630, China

## Abstract

Sepsis-induced myocardial dysfunction increases mortality in sepsis, yet the underlying mechanism is unclear. Brain-derived neurotrophic factor (BDNF) has been found to enhance cardiomyocyte function, but whether BDNF has a beneficial effect against septic myocardial dysfunction is unknown. Septic shock was induced by cecal ligation and puncture (CLP). BDNF was expressed in primary cardiomyocytes, and its expression was significantly reduced after sepsis. In rats with sepsis, a sharp decline in survival was observed after CLP, with significantly reduced cardiac BDNF expression, enhanced myocardial fibrosis, elevated oxidative stress, increased myocardial apoptosis, and decreased endothelial nitric oxide (NO) synthase (eNOS) and NO. Supplementation with recombined BDNF protein (rhBDNF) enhanced myocardial BDNF and increased survival rate with improved cardiac function, reduced oxidative stress, and myocardial apoptosis, which were associated with increased eNOS expression, NO production, and Trk-B, a BDNF receptor. Pretreatment with NOS inhibitor, N (omega)-nitro-L-arginine methyl ester, abolished the abovementioned BDNF cardioprotective effects without affecting BDNF and Trk-B. It is concluded that BDNF protects the heart against septic cardiac dysfunction by reducing oxidative stress and apoptosis via Trk-B, and it does so through activation of eNOS/NO pathway. These findings provide a new treatment strategy for sepsis-induced myocardial dysfunction.

## 1. Introduction

Sepsis is identified as a systemic deleterious inflammatory response to infection or injury [1]. The resulting severity of sepsis and septic shock is associated with high mortality rate, which mainly results from dysfunction and failure of vital organs [2]. In particular, cardiac dysfunction is the leading cause of death in patients with sepsis [3–6]. Therefore, patient's ability to recover from septic myocardial dysfunction becomes a key predictor of survival [7]. Despite over three decades of clinical and basic research on sepsis-induced myocardial dysfunction, current understanding of the pathophysiology of myocardial dysfunction in sepsis is limited and effective therapies are lacking for this disorder [8].

Septic myocardial dysfunction is complicated and multifactorial, which involved persistent inflammation-induced microlesions of endothelium and endocardium, alterations in intracellular calcium homeostasis, contractile dysfunction of the heart, increase of reactive oxygen species (ROS), and apoptosis [9, 10]. Besides cardiac contractility dysfunction, oxidative stress has been considered to play a critical role in the progression of sepsis-induced myocardial dysfunction [11–13]. Ample evidence has demonstrated protective effects of antioxidant facilitation in sepsis, and reinforcement of myocardium endogenous antioxidant defense attenuates cardiac oxidative stress and preserves contractile reserve [11, 14–16].

Brain-derived neurotrophic factor (BDNF) is a growth factor that is widely expressed in the nervous system. Low serum BDNF level was found in patients with chronic heart failure [17] and was positively correlated with heart failure severity [18], indicating that BDNF may play a role in heart failure. Indeed, previous study demonstrated that an intact BDNF/Trk-B (tropomyosin-related kinase receptor B, a BDNF receptor) signaling was required for enhancing normal cardiac calcium cycling, maintaining optimal cardiac contraction and relaxation, while loss or impairment of BDNF/Trk-B may underlie the pathogenesis of myocardial dysfunction in acute or chronic heart diseases [19]. Moreover, studies showed that BDNF can increase nitric oxide (NO) level in neuronal cells while endothelial NO synthase (eNOS) protects neurons through the BDNF/Trk-B pathway during brain ischemic injury [20–23]. Given that eNOS-derived NO induces myofibril relaxation, which is considered important in protection against septic myocardial dysfunction [24, 25] and that eNOS-derived NO represents one of the most important protective molecule that fights a wide range of cardiovascular disease resulting from fibrosis to oxidative stress [26], the properties of BDNF (e.g., enhancing calcium cycling and increasing NO) provide a clue that BDNF may protect the heart against septic cardiac dysfunction by increasing NO and the subsequent reduction of oxidative stress. However, whether BDNF can stimulate NO to confer cardioprotection in the myocardium and whether BDNF confers cardioprotective effect by reducing oxidative stress remain unknown. We, therefore, hypothesized that BDNF attenuates septic cardiac dysfunction by reducing oxidative stress and its dose so by enhancing NO.

## 2. Materials and Methods

### 2.1. Animals

The study was approved by the Medical Ethics Committee of The Second Xiangya Hospital of Central South University in Changsha, P.R. China and followed the NIH guidelines (Guide for the care and use of laboratory animals). Specific pathogen-free adult male Sprague-Dawley (SD) rats (Aged 6–8 weeks; weighted 180–200 g, Changsha, China) were housed under identical conditions (room temperature at 25°C, 50 ± 10% relative humidity, and 12 hour light-dark cycle) and had free access to a standard rodent diet and water. The animal experiments were performed according to the guidelines for the care and use of animals established by Central South University.

### 2.2. Animal Model of Sepsis

The sepsis model of rats with cecal ligation and puncture (CLP) was obtained as described [27, 28]. The rats were positioned on a homoeothermic heating pad in order to maintain body temperature about 37°C. Under complete anesthetization by inhaling 1–3% isoflurane and 40% oxygen during surgery, a 3 cm midline laparotomy on the anterior abdomen was made in the rats. The cecum was exposed and ligated using a 3–0 silk suture just below the ileocecal valve in order to avoid intestinal obstruction. The cecum was punctured twice on the anti-mesenteric border with a 16-gauge (1.65 mm diameter) needle and returned to the abdominal cavity. The incision was then closed with 4-0 silk suture. Each rat received normal saline (4 mL/100 g) by subcutaneous injection immediately after CLP. The rats were then returned to room air and had free access to water after CLP. Sham-operated rats underwent the same above surgical procedure, in addition to the cecum was neither ligated nor punctured.

**Figure 1:**
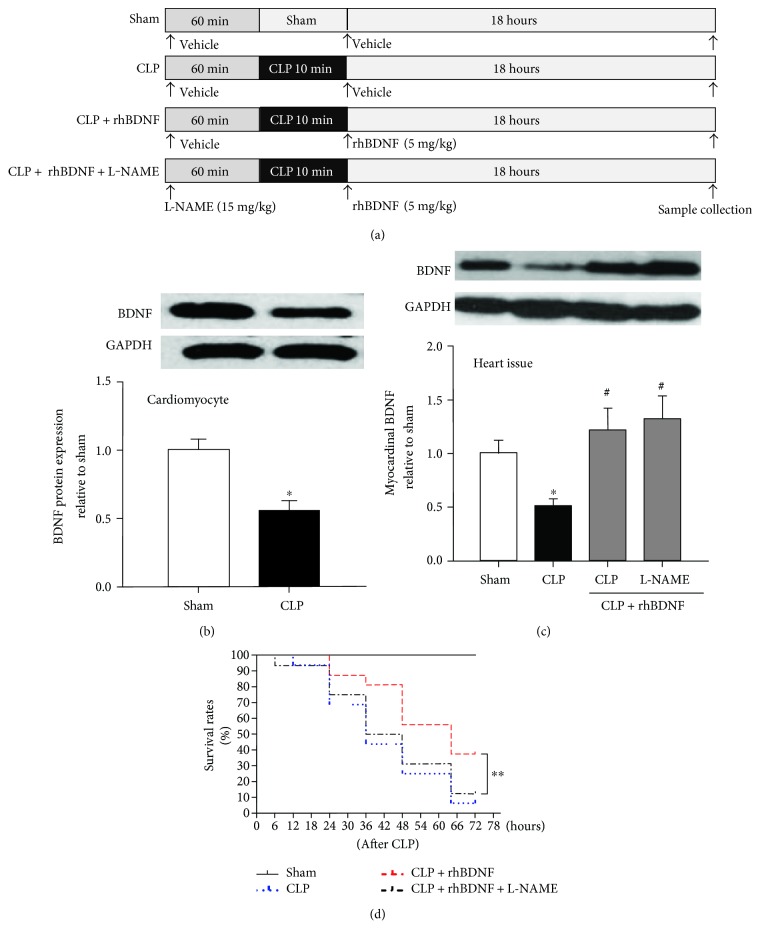
BDNF expressed in cardiomyocytes was reduced after septic shock, and supplementation of BDNF improved survival rate in rats subjected to septic shock. (a) Schematic diagram of the protocol. (b) BDNF protein expression in isolated cardiomyocytes. (c) BDNF protein expression in heart tissues. (d) Survival rates in rats subjected to septic shock. Data are mean ± SEM, with *n* = 8 animals per group. ^∗^*P* < 0.05 versus sham; ^#^*P* < 0.05 versus CLP; ^∗∗^*P* < 0.01. CLP: cecal ligation and puncture; rhBDNF: recombined BDNF protein.

Rats were divided into four groups (*n* = 8  per  group) as illustrated in Figure 1(a): the control (vehicle + sham) group, vehicle + CLP group, CLP + rhBDNF (5 mg/kg, i.v., Peprotech, Rocky Hill, USA) group, and CLP + rhBDNF + L-NAME (15 mg/kg, i.v., Sigma, USA). The schematic diagram of the protocol was shown in Figure 1(a). At the late, hypodynamic stage of sepsis (i.e., 18 hours after CLP) or sham operation, rats were anesthetized by inhaling 1–3% isoflurane and 40% oxygen. A catheter filled with heparin saline (500 U/mL) was inserted into the left ventricle from the right carotid artery to measure mean arterial blood pressure (MABP) and left ventricular (LV) pressure. Maximal LV pressure development (LVdp/dtmax), LV endsystolic pressure, LV end-diastolic pressure (LVEDP), and heart rate were recorded by using a Powerlab (4S, Australia).

### 2.3. Adult Rat Ventricular Cardiomyocyte Isolation

Calcium-tolerant cardiomyocytes were isolated from rat ventricles via a modified method as previously described [29]. Rats receiving sham operation or CLP were sacrificed with an intraperitoneal injection of overdose sodium pentobarbital (220 mg/kg) and heparinized. The hearts were rapidly removed and mounted on a Langendorff perfusion apparatus and proceeded to cardiomyocytes isolation as descried [30]. Cells isolated from a single rat heart were plated onto Matrigel-coated culture dishes and allowed to recover for 3 hours. Cultured ventricular cardiomyocytes were incubated in Medium 199 (Gibco, Grand Island, NY) at 37°C for 2 hours then snap-frozen in liquid nitrogen for future analysis.

### 2.4. Samples Collected and Histopathology Analysis

Rats were killed using carbon dioxide inhalation smoothing method at 18 hours after CLP surgery, and left ventricular myocardial tissues were collected. Tissue sections of the myocardium were stained with hematoxylin-eosin (H&E) staining as previously described [29], and morphological changes were evaluated using light microscopy at a magnification of 400x.

### 2.5. Masson's Trichrome Staining

Paraffin-embedded tissues were cut into 5 *μ*m of section for Masson's trichrome staining (Beijing Rocchi Biotechnology Co. Ltd., China) in strict accordance with the kit experimental steps. Leica Image Processing and Analysis System (Leica Microsystems Digital Imaging, Cambridge, UK) were used for image acquisition and semi-quantitative analysis of the results of Masson staining. Five visual fields were randomly selected for each rat to calculate the average index number of myocardial collagen volume fraction.

### 2.6. ELISA Kit for 15-F2t-Isoprostane

Oxidative stress biomarker 15-F2t-isoprostane in myocardial tissue was measured by using commercially available 15-F2t-isoprostane ELISA kit (Cayman, USA), in strict accordance with the kit manufacturer's instructions as described [30]. All samples were measured in triplicate.

### 2.7. Measurement of Superoxide Dismutase (SOD)

The myocardial tissue was collected, and SOD activities were measured using commercially available kits, according to the manufacturer's instructions (Nanjing Keygen Biotech. Co. Ltd., Nanjing, China).

### 2.8. Measurement of Myocardial Levels of Nitric Oxide

As the stable end products of nitric oxide (NO), nitrites (NO_2_^−^), and nitrates (NO_3_^−^) were determined in the rats' ventricular tissue by a NO colorimetric assay kit (BioVision, Inc., California).

### 2.9. TUNEL Staining

Myocardial apoptosis was analyzed using a terminal deoxynucleotidyl transferase dUTP nick-end labeling (TUNEL) assay using a commercial kit (Roche Diagnostics, Indianapolis, IN, USA) according to a previously described methodology [29].

### 2.10. Immunohistochemistry (IHC) Analysis

Paraffin-embedded tissues were cut into 4 *μ*m-thick sections, followed by antigen unmasking process, and incubated overnight at 4°C with 4-HNE rabbit antibody (1/200 dilution; Abcam, Cambridge, UK) or eNOS mouse antibody (1/100 dilution; Millipore, Billerica, MA, USA). Phosphate buffered saline replaced the primary antibody as a negative control. The subsequent detection was use of anti-rabbit or mouse immunohistochemistry assay kit (DAKO, Carpentaria, CA, USA) as a chromogen for visualization. Finally, hematoxylin was used to counterstain the nuclei. All the slides were viewed and photographed under microscope combined with a digital camera (Leica Microsystems Digital Imaging, Cambridge, UK).

### 2.11. Immunofluorescence Analysis

4 *μ*m thick paraffin-embedded sections were incubated overnight with TrkB rabbit antibody (1/200 dilution; Santa, Cruz, CA, USA) after subjecting to standard procedure for dewaxing, blocking endogenous peroxidase, and exposing antigenic sites. A FITC-conjugated goat anti-rabbit antibody (1 : 100; Zhongshan Gold Bridge) was used to detect the above primary antibody. Slides were detected using a fluorescence microscope (Leica Microsystems Digital Imaging, Cambridge, UK). We chose five 40x magnification fields per tissue section at random, and two independent blinded observers obtained the mean area values of positive signals for final analysis by using the Image-Pro Plus 6.0 software.

### 2.12. Western Blot

Myocardial tissues were grounded and homogenized with lysis solution. After sonication, the lysates were centrifuged, and the proteins were separated using electrophoresis and transformation to polyvinylidene fluoride (PVDF) membranes. After being blocked with 5% skim milk in Tris-buffered saline (TBS) for 2 hours at room temperature, the membrane was incubated with primary antibodies against BDNF rabbit antibody (1/1000 dilution; Abcam, Cambridge, UK), Bax, Bcl-2, caspase-3, cleaved caspase-3 rabbit antibody (1/1000 dilution; Cell signaling Tecnology, USA), and GAPDH mouse antibody (1/1000 dilution; Cell signaling Tecnology, USA) overnight at 4°C, washed three times with TBST, and then incubated with horseradish peroxidase-conjugated secondary antibody for 1 hour at 37°C. The blots were imaged using AlphaView system (Cell Biosciences, Santa Clara, CA, USA) and quantified using the Image J 1.48 software (National Institutes of Health).

### 2.13. Evaluation of Survival Rate

The rats (*n* = 16  *per*  group) receiving the same protocols were used to assess survival rates. The rats in each group had free access to food and water and were kept under pathogen-free conditions. Animals were monitored via video recording, and the survival rate was evaluated within 3 days in each group.

### 2.14. Statistical Analysis

All data were described as mean ± standard error of measurement (SEM) and analyzed using GraphPad Prism 6. One-way ANOVA was selected to compare data of more than two groups, and multiple comparisons were performed by Tukey's Honestly Significant Difference test. *P* < 0.05 was considered statistically significant.

## 3. Result

### 3.1. BDNF Expression in Cardiomyocytes Was Reduced after Septic Shock

Previous studies have shown that BDNF is expressed in many types of tissues or cells [31], but it is unknown whether BDNF is expressed in cardiomyocytes. In primary cardiomyocytes isolated from rats, we showed that BDNF was expressed in cardiomyocytes and was reduced after CLP (Figure 1(b)). Similar trends of change of BDNF protein expression was observed in myocardial tissue in rats subjected to CLP (Figure 1(c)). We then tried to restore myocardial BDNF by intraperitoneal administration of recombined BDNF; as shown in Figure 1(c), BDNF administration significantly increased myocardial BDNF protein expression after CLP.

### 3.2. Improvement of Cardiac Function by BDNF in Septic Shock Rats That Was Aggravated by L-NAME

As shown in Table 1, cardiac function in rats after CLP was impaired, demonstrated by reduction of MABP, dP/dt_max_, dP/dt_min_, and LVSP and increase of LVEDP compared to that in sham, which was eradicated by supplementation of BDNF. However, these beneficial effects of BDNF were reversed by L-NAME pretreatment evidenced by increase of MABP, dP/dt_max_, dP/dt_min_, and LVSP and decrease of LVEDP (*P* < 0.05 versus CLP + rhBDNF).

### 3.3. Increased Rats' Survival Rate by BDNF after Septic Shock That Was Reversed by L-NAME

As shown in Figure 1(d), survival rate dropped about 10% in rats subjected to CLP within 12 hours after septic shock and continued to decline sharply starting from 24 hours after injury, reaching almost 0% by 72 hours after CLP (Figure 1(d)). Intravenous administration of BDNF extended life time, and increased survival rate that first drop of survival rate was observed at 24 hours after CLP and about 40% of animals survived by 72 hours after injury (Figure 1(d)). This effect of BDNF was abolished by L-NAME (Figure 1(d)).

### 3.4. Reduction of Cardiac Hypertrophy by BDNF in Septic Shock Rats That Was Reversed by L-NAME

**Figure 2:**
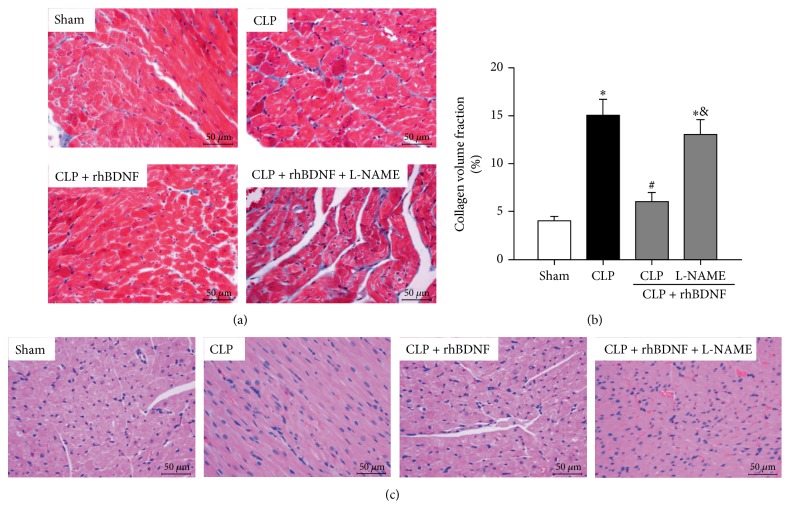
BDNF reduced cardiac hypertrophy in septic shock rats that was attenuated by L-NAME. (a and b) Myocardial fibrosis content assessed by Masson's trichrome staining (400x magnification) and quantitation. (c) The cell size visualization by H&E staining (400x magnification). Data are mean ± SEM, with *n* = 8 animals per group. ^∗^*P* < 0.05 versus sham; ^#^*P* < 0.05 versus CLP; ^&^*P* < 0.05 versus CLP + rhBDNF. CLP: cecal ligation and puncture; rhBDNF: recombined BDNF protein.

Myocardial fibrosis is a hallmark of cardiac hypertrophy [32]. Myocardium was stained with Masson's trichrome (Figures 2(a) and 2(b) and H&E (Figure 2(c)) to identify cardiac fibrosis and cardiomyocyte morphology, respectively, after CLP. As shown in Figure 2, collagen volume was significantly increased after CLP (CLP versus sham, *P* < 0.05), which was reduced by BDNF (CLP + rhBDNF versus CLP, *P* < 0.05). Pretreatment with L-NAME reversed the effect of BDNF manifested as profound increase of cardiac collagen volume in the CLP + rhBDNF + L-NAME group compared to CLP + rhBDNF group.

### 3.5. Attenuation of Myocardial Oxidative Stress by BDNF in Septic Shock Rats That Was Abolished by L-NAME

**Figure 3:**
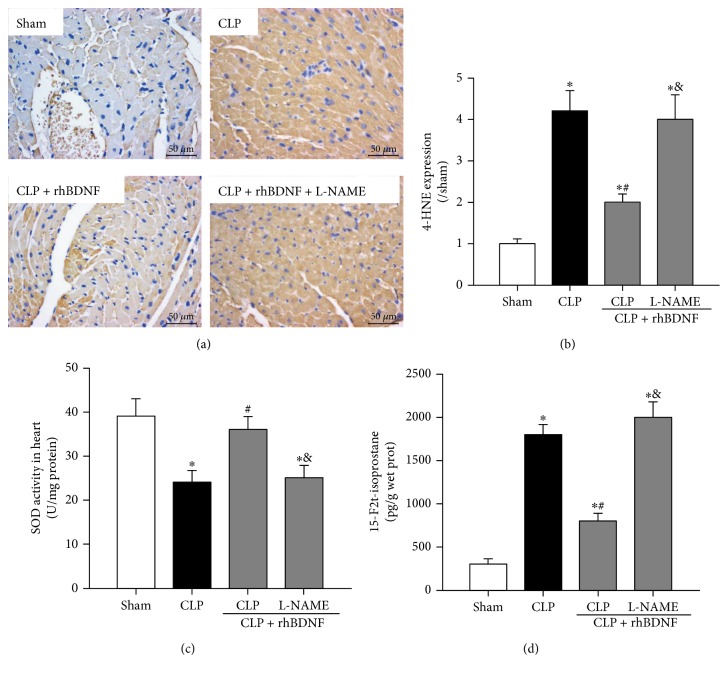
BDNF attenuated myocardial oxidative stress in septic shock rats that was abolished by L-NAME. (a and b) Visualization and quantitation of 4-HNE in heart tissue. (c) Superoxide dismutase (SOD) activity in heart tissue. (d) Free 15-F2t-isoprostane in heart tissue was measured by using an enzyme-linked immunoassay kit. Data are mean ± SEM, with *n* = 8 animals per group. ^∗^*P* < 0.05 versus sham; ^#^*P* < 0.05 versus CLP; ^&^*P* < 0.05 versus CLP + rhBDNF. CLP: cecal ligation and puncture; rhBDNF: recombined BDNF protein.

Myocardial level of 4-HNE, a marker of lipid peroxidation, and cardiac level of 15-F2t-isoprostane, a specific index of ROS-induced oxidative stress, were significantly increased after CLP that were associated with reduced superoxide radical scavenging enzymatic activity of SOD (Figures 3(a), 3(b), 3(c), and 3(d)). All these changes were attenuated by BDNF. However, these beneficial effects of BDNF were reduced by L-NAME pretreatment (Figures 3(a), 3(b), 3(c), and 3(d)).

### 3.6. BDNF Reduced Cardiomyocyte Apoptosis after Septic Shock That Was Reduced by L-NAME

**Figure 4:**
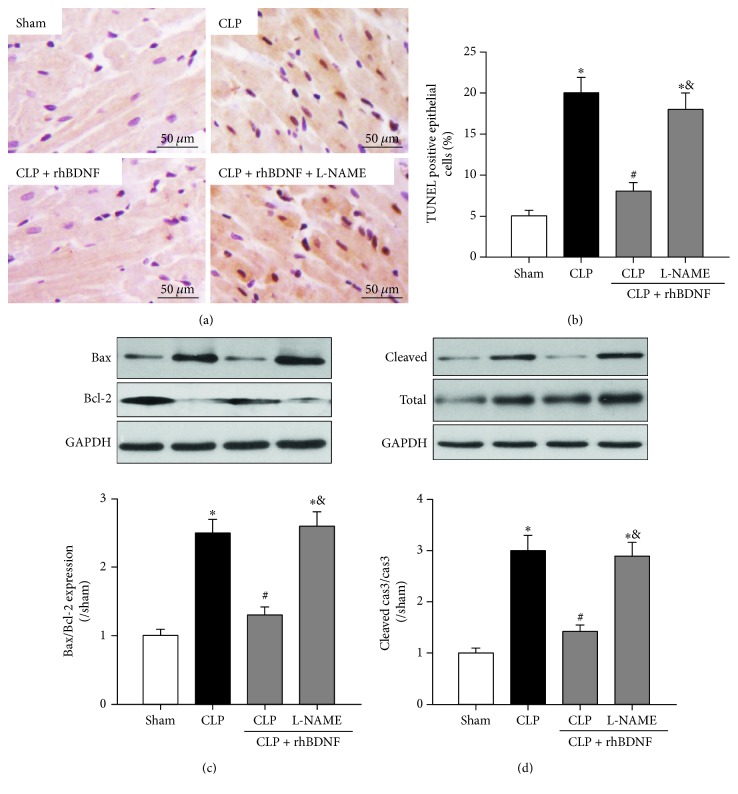
BDNF reduced cardiomyocyte apoptosis after septic shock that was reduced by L-NAME. (a and b) Visualization (400x magnification) and quantitation of TUNEL positive cells in heart tissue. (c) Bax and Bcl-2 protein expression. (d) Total and cleaved caspase-3 protein expression. Data are mean ± SEM, with *n* = 8 animals per group. ^∗^*P* < 0.05 versus sham; ^#^*P* < 0.05 versus CLP; ^&^*P* < 0.05 versus CLP + rhBDNF. CLP: cecal ligation and puncture; rhBDNF: recombined BDNF protein.

Myocardial cell apoptosis was significantly enhanced after CLP evidenced by increased number of TUNEL-positive cells (Figures 4(a) and 4(b)), elevated Bax to Bcl-2 ratio (Figure 4(c)), and upregulated cleaved caspase-3 protein expression (Figure 4(d)). These alternations were reduced by supplementation of BDNF, while these antiapoptotic effects of BDNF were abolished by L-NAME (Figures 4(a), 4(b), 4(c), and 4(d)).

### 3.7. BDNF Increased Induction of eNOS-Derived NO and Enhanced Trk-B in Septic Shock Rats

**Figure 5:**
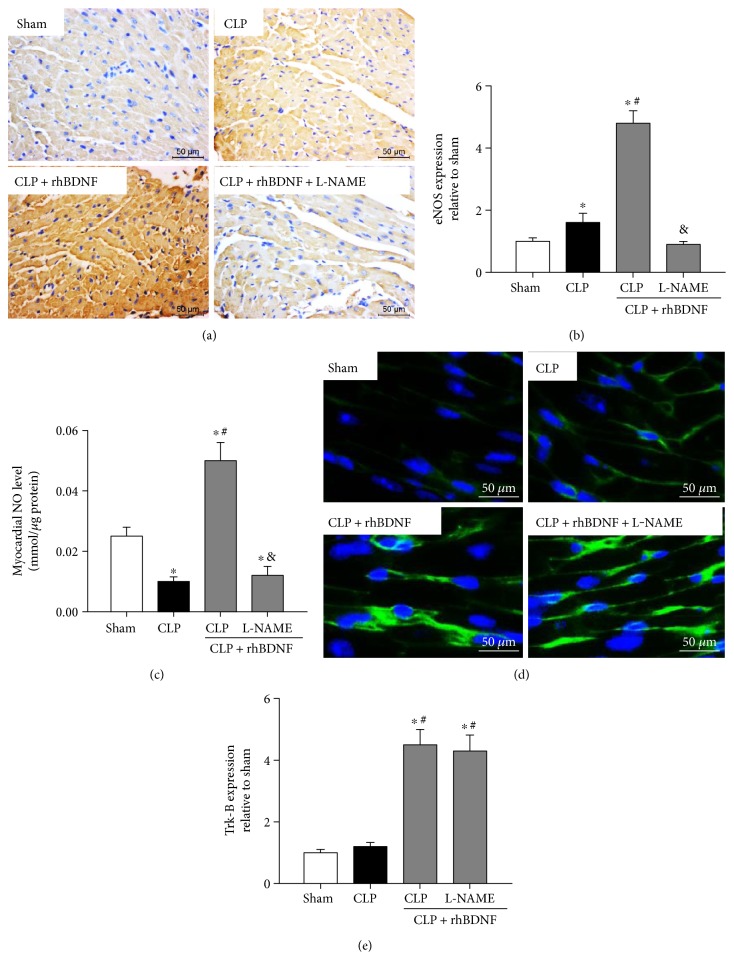
BDNF increased induction of eNOS-derived NO and enhanced Trk-B in septic shock rats. (a and b) Visualization (400x magnification) and quantitation of eNOS protein expression in heart tissue. (c) Nitric oxide (NO) production in heart tissue. (d and e) Visualization (400x magnification) and quantitation of Trk-B protein expression. Data are mean ± SEM, with *n* = 8 animals per group. ^∗^*P* < 0.05 versus sham; ^#^*P* < 0.05 versus CLP; ^&^*P* < 0.05 versus CLP + rhBDNF. CLP: cecal ligation and puncture; rhBDNF: recombined BDNF protein.

In Figures 5(a), 5(b), and 5(c), after CLP, expression of eNOS was significantly increased while NO level was reduced significantly. Although supplementation of BDNF further upregulated eNOS protein expression and increased NO production by more than twice of the amount in CLP groups, these effects of BDNF were abolished by L-NAME (Figures 5(a), 5(b), and 5(c)). Trk-B is a receptor of BDNF that is highly expressed in cardiomyocytes [19]. As shown in Figures 5(d) and 5(e), CLP has no effect on Trk-B protein expression. BDNF supplementation significantly increased Trk-B protein expression, while pretreatment of L-NAME has no impact on BDNF-induced increase of Trk-B protein expression (Figures 5(d) and 5(e)).

## 4. Discussion

With regard to the high mortality rate in sepsis, one of the key predictors of survival is the patients' ability to recover from septic myocardial dysfunction [7]. Our study found that supplementation of BDNF after septic shock is effective in protecting the hearts against septic cardiac dysfunction. To our knowledge, this is the first study which provided direct proof that BDNF is expressed in cardiomyocyte and that cardiomyocytes BDNF is reduced after sepsis. We further demonstrated that, in CLP-induced sepsis, myocardial BDNF was reduced that was associated with impaired cardiac function, increased myocardium fibrosis, elevated cardiomyocyte apoptosis, reduced NO production, and increased oxidative stress. Supplementation of BDNF after CLP boosted BDNF level in myocardium and rescued cardiac dysfunction, enhanced NO and eNOS, upregulated Trk-B protein expression, attenuated oxidative stress, and, eventually, improved animal survival. All these effects of BDNF were abolished by NOS inhibition, suggesting BDNF conferred myocardial protection through the stimulation of eNOS, resulting in increased NO level, and thus reduced oxidative stress and cardiac fibrosis thereby improving postseptic cardiac functional recovery and survival. These findings provide evidence that effective treatment targeting BDNF may facilitate the recovery of septic myocardial dysfunction.

BDNF, a pleiotropic neurotrophin, is best characterized for its ability to promote neurogenesis by stimulation of its receptor, Trk-B, in neuronal cells [33, 34]. While BDNF/Trk-B is identified throughout the central and peripheral nervous systems, they also found it in various nonneuronal tissues such as endothelial cells in the heart, muscle, and vasculature as well as smooth and skeletal muscle cells, enhancing cell survival and function [35, 36]. However, little is known about its role in myocardial physiology and pathology. Recent study showed that BDNF/Trk-B signaling is required for optimal cardiac contraction and relaxation and that loss or impairment of BDNF/Trk-B function may lead to myocardial dysfunction [19]. The present study in isolated primary cardiomyocytes provided direct evidence that BDNF is constitutively expressed in cardiomyocytes, the predominant cell type in the heart. This together with the previous study shows that Trk-B is highly expressed in the heart indicating that BDNF may play a critical role in cardiomyocyte survival and functional maintenance in an autocrine manner.

In myocardium from CLP-induced septic shock rats, cardiac BDNF level was significantly reduced accompanied with increased cardiomyocyte apoptosis and enhanced oxidative stress, which were associated with cardiac dysfunction and increased mortality rate. All these were reversed by BDNF supplementation. Given that apoptosis and the subsequent cell loss, which were stimulated by oxidative stress, have been considered a driving force and the major mechanism in sepsis-induced myocardial dysfunction [37], the observed cardioprotective effects of BDNF in the hearts from septic shock rats may act through reducing oxidative stress and the subsequent reduction of cardiomyocytes apoptosis. Our findings were similar to the study by Hang et al. which showed that BDNF by downregulating microRNA-195 inhibited cardiac apoptosis and attenuated myocardial ischemia reperfusion injury in rats [38]. In the current study, we further demonstrated that the abovementioned beneficial effects of BDNF were mainly through NOS system.

Recent studies identified crosstalk between BDNF and NO in neuronal cell [20, 22]. In the current study, we provided additional evidence that similar regulation of NO via BDNF also present in cardiomyocyte. It is well acknowledged that NO is critical in the pathogenesis of sepsis and that eNOS-mediated NO production is important in maintaining proper function of the cardiovascular system, while under pathological conditions (i.e., sepsis and ischemia reperfusion injury), deregulated or excessive release of NO by inducible NO synthase (iNOS) contributes substantially to cardiac dysfunction [26, 39]. Loss or malfunction of eNOS resulting in low level of NO bioavailability and the subsequent increase of oxidative stress has been considered an important component of septic myocardial dysfunction and development of heart failure [25, 40]. In our study, despite compensatory increase of eNOS, which is in line with the reported transient increase of eNOS during acute phase of pathological condition [41], NO level remained low, which was associated with cardiac dysfunction. BDNF supplementation restored eNOS and NO and attenuated cardiac dysfunction; all these were abolished by NOS inhibition, highlighting the importance of NO in BDNF cardioprotection in sepsis. However, the source of this BDNF-induced NO production is still unknown. Studies have shown that once expressed iNOS produces large quantities of NO over a long period of time in response to proinflammatory cytokines during sepsis, which adversely affects myocardial contractile function and leads to myocardial depression [42]. Yet, the time course of iNOS induction seems to be tissue- and specie-dependent. Elevated level of iNOS was found in the lung, spleen, and liver within 4 hours after septic stimulation in both dog and rat, but increase of iNOS in the heart was observed 6 hours afterwards in dog while no upregulation of iNOS was detected at 24 hours in rat after initiation of sepsis [43, 44]. Hence, our data suggests that in our CLP-induced sepsis model in rat, eNOS rather than iNOS takes place to maintain regulated NO production during early phase of sepsis (18 hours after CLP in our septic model). As a result, after CLP, the low level of NO, which was eNOS-derived, was associated with increased lipid peroxidation and ROS-induced oxidative stress and decreased SOD activity in the myocardium.

It is of notice that recent studies suggest that other types of cell death such as autophagy and necroptosis also play roles in cardiac hypertrophy [45, 46]. In the current study, although we provided evidences that BDNF conferred cardioprotective effects against cardiac hypertrophy in sepsis rats was mainly through reducing cardiomyocytes apoptosis, we could not rule out the possibility that other types of cell death such as autophagy and necroptosis may also play roles in these BDNF-induced cardioprotection; further investigation is needed to address the roles of other types of cell death in BDNF-induced cardioprotection. Moreover, in our study, BDNF was given after the onset of CLP, whether or not pretreatment of BDNF also exerts cardioprotection in sepsis remains known, which needs for further investigation.

## 5. Conclusions

To our knowledge, our study for the first time demonstrated the innate expression of BDNF in cardiomyocyte and the cardioprotective role of BDNF in protecting hearts against sepsis-induced cardiac dysfunction and animal death. Upregulation of eNOS and subsequent induction of NO represent a major mechanism where BDNF reduces oxidative stress and decreases myocardial apoptosis and eventually attenuates cardiac dysfunction and improves animal survival in sepsis.

## Figures and Tables

**Table 1:** BDNF improved cardiac function in septic shock rats that was reduced by L-NAME.

Group	HR (beats/min)	MABP (mmHg)	LV dP/dt_max_ (mmHg/s)	LV dP/dt_min_ (mmHg/s)	LVEDP (mmHg)	LVSP (mmHg)
Sham	430 ± 52	110.52 ± 12.64	6682 ± 646	−4818 ± 356	11.22 ± 4.86	130.24 ± 11.46
CLP	460 ± 42	68.46 ± 15.38^∗^	3980 ± 386^∗^	−2860 ± 246^∗^	19.82 ± 5.02^∗^	89.74 ± 9.12^∗^
CLP + BDNF	446 ± 54	96.86 ± 12.12^#^	6024 ± 562^#^	−4328 ± 302^#^	13.02 ± 4.06^#^	124.68 ± 12.48^#^
CLP + BDNF + L-NAME	458 ± 50	70.02 ± 10.46^&^	4126 ± 428^&^	−3022 ± 286^&^	18.98 ± 5.82^&^	94.56 ± 10.06^&^

HR: heart rate; MABP: mean arterial blood pressure; LV dP/dt_max_: left ventricular (LV) maximal pressure development; LV dP/dt_min_: left ventricular (LV) minimal pressure development; LVEDP: left ventricular end-diastolic pressure; LVSP: left ventricular systolic pressure. Data are mean ± SEM. ^∗^*P* < 0.05 versus control group, ^#^*P* < 0.05 versus CLP group, ^&^*P* < 0.05 versus CLP +  rhBDNF. CLP: cecal ligation and puncture; rhBDNF: recombined BDNF protein.
